# When Treatment Destabilises - Managing Ultra-Rapid Cycling Bipolar Affective Disorder Complicated by Hypothyroidism and Antidepressant Sensitivity

**DOI:** 10.1192/bjo.2026.11882

**Published:** 2026-06-30

**Authors:** Gayathri Rangith

**Affiliations:** Cygnet Health Care, Oldbury, United Kingdom

## Abstract

**Aims:**

Ultra-rapid cycling bipolar affective disorder (BPAD), defined by mood episodes occurring within weeks rather than months, is associated with significant morbidity and limited evidence-based guidance. Management becomes particularly challenging in women with pre-existing biological vulnerability factors such as hypothyroidism and pronounced sensitivity to antidepressants. This case highlights the complexity of balancing suicide risk, patient preference, and treatment-related mood destabilisation in ultra-rapid cycling BPAD.

**Methods:**

We describe the case of a woman with a diagnosis of ultra-rapid cycling BPAD and a history of multiple prior admissions for mood instability. She had pre-existing hypothyroidism, neurodevelopmental comorbidity, and a past history of substance misuse, with no active substance use during admission. The patient experienced severe bipolar depressive episodes associated with high suicide risk, for which duloxetine was prescribed. However, even minor antidepressant dose changes were associated with mood elevation.

Lithium was optimised and maintained within the therapeutic range throughout admission, alongside initiation of clozapine for mood stabilisation. Following an inadvertent antidepressant dose escalation, the patient developed emerging manic symptoms, prompting urgent multidisciplinary medication review. Hypothyroidism management was concurrently reassessed, revealing suboptimal levothyroxine administration due to incorrect timing with food and other medications. Targeted education for the patient and staff was implemented, alongside dose escalation and regular thyroid function monitoring.

**Results:**

This case demonstrates pronounced antidepressant sensitivity in ultra-rapid cycling BPAD, with dose variability precipitating mood destabilisation even at low doses. Hypothyroidism represented a potentially modifiable biological vulnerability factor, with impaired medication absorption contributing to affective instability. A key clinical paradox emerged in which treatment required to mitigate suicide risk simultaneously increased the risk of manic relapse.

Shared decision making was central to management. Despite guideline based concerns regarding antidepressant use in rapid cycling BPAD, a consensus decision which was supported by wider acute consultant discussion and independent second opinion was made to continue antidepressant treatment at a reduced dose for a limited period, with close monitoring. Diagnostic clarity and coordinated multidisciplinary working were essential in maintaining therapeutic alliance and managing risk.

**Conclusion:**

Ultra-rapid cycling BPAD necessitates highly individualised care. Antidepressants may destabilise mood even at low doses in biologically vulnerable patients, particularly women with comorbid hypothyroidism. Optimisation of physical health conditions, including attention to medication administration practices, can be clinically significant. This case underscores the importance of balancing guideline recommendations with patient-centred decision-making in complex, high-risk presentations.

